# Total hip arthroplasty in hereditary multiple exostoses with secondary osteoarthritis: A case report

**DOI:** 10.1097/MD.0000000000018175

**Published:** 2019-11-27

**Authors:** Woo Jong Kim, Jong Seok Park, Sung Hun Won, Hong Seop Lee, Dhong Won Lee, Byung-Woong Jang, Jae Jun Lee, Yong Cheol Hong, Jin Ku Kang, Ricky Wibowo, Chang Hyun Kim

**Affiliations:** a Department of Orthopedic Surgery, Soonchunhyang University Hospital Cheonan, Cheonan; b Department of Orthopedic Surgery, Soonchunhyang University Hospital Seoul; c Department of Foot and Ankle Surgery, Nowon Eulji Medical Center, Eulji Uiversity; d Department of Orthopedic Surgery, Konkuk University Medical Center, Seoul; e Department of Anesthesia, Soonchunhyang University Hospital Cheonan, Cheonan, South Korea; f Department of Orthopedics and Traumatology Faculty of Medicine Universitas Padjadjaran Bandung, West Java, Indonesia.

**Keywords:** hereditary multiple exostoses, hip joint, osteoarthritis, replacement arthroplasty

## Abstract

**Rationale:**

Hereditary multiple exostoses (HME) is an autosomal dominant disease that causes multiple exostoses throughout the body. It usually occurs around the metaphysis of the long bones, and when it involves the hip, symptoms arise due to deformity and the mass effect. If the lesion does not involve the joint or is not associated with arthritis, symptoms can be relieved by surgical excision of the osteochondroma. However, if secondary osteoarthritis (OA) or subluxation of the joint has progressed, joint replacement arthroplasty should be considered.

**Patient concerns:**

A 57-year-old woman with HME visited our outpatient department with severe right hip pain. She complained of difficulty walking and severe discomfort during activities of daily living. She was short in stature and had a family history of HME.

**Diagnosis:**

A physical examination revealed limited motion in the hip joint and a limb length discrepancy. Plain radiography and a computed tomography scan revealed huge osteochondromas on bilateral proximal femurs and advanced OA with subluxation of the right hip joint.

**Interventions:**

Cementless total hip arthroplasty of the right hip joint via the modified posterolateral approach was done.

**Outcomes:**

The patient showed good clinical scores and functional improvement at the 2-year follow-up.

**Lessons:**

Total hip arthroplasty for an anatomically deformed joint is technically difficult, and there are many factors to consider that can make surgeons reluctant to use this modality. However, with careful preparation, arthroplasty is a good surgical option for symptomatic and functional recovery in HME patients with hip joint involvement.

## 1 Introduction

Hereditary multiple exostoses (HME) is an autosomal dominant disease in which multiple osteochondromas develop next to the growth plates of the long bones, ribs, hips, and vertebrae.^[1,2]^ Small exostoses rarely cause symptoms because of their deep anatomical locations, but surgical correction is required if they become large or cause a deformity or neurovascular complication. The timing and method of therapeutic intervention must be considered carefully because about 2% of HME patients have the potential for experiencing malignant transformations, such as chondrosarcoma or osteosarcoma, and HME itself affects quality of life of the patients.^[2,3]^

The incidence of exostoses around the hip in HME patients has been reported to be 30% to 90%,^[4–7]^ but bilateral hip lesions in patients with HME are rare, and the lesions are associated with premature osteoarthritis (OA) of the hip.^[8]^ In addition, osteochondromas around the hip cause dysplasia of the proximal femur and acetabulum, and compress adjacent soft tissue directly, causing limitations in motion, joint subluxation, and persistent pain. If only impingement and pain caused by a lesion are present, resection of the tumor should relieve the pain,^[9]^ but total hip arthroplasty is an effective single-stage intervention in the case of advanced OA accompanied by a limb length discrepancy. However, distorted anatomy of the hip joint resulting from an expanded metaphysis of the proximal femur and a coxa valga deformity can make the surgery technically difficult. We present a case of HME with secondary OA of the hip joint treated successfully by total hip arthroplasty.

## 2 Case description

This case report was approved by the Institutional Review Board of Soonchunhyang University Hospital, and the patient provided written informed consent for publication of the report and accompanying images. A 57-year-old female with HME visited our outpatient department with severe right hip pain for the past 3 years. The pain was not severe at the early stage but had gradually progressed, leading to difficulty walking and severe discomfort during daily living. She had multiple bony humps over her body and had a family history of similar deformities (in her daughter and grandchildren). She had no other relevant medical history, but was short in stature, at 145 cm.

A physical examination revealed a waddling gait, and there were multiple irregular bony protrusions around the hips, knees, and ankle joints. The range of motion (ROM) of the hip joint was 25° of hip flexion, 10° of external rotation, and 10° of internal rotation on the right side, and 90° of hip flexion, 20° of external rotation, and 15° of internal rotation on the left side. No disturbance in the blood circulation and no neurological abnormalities were observed in either lower extremity. The right lower extremity was 2.4 cm longer than the left.

**Figure 1:**
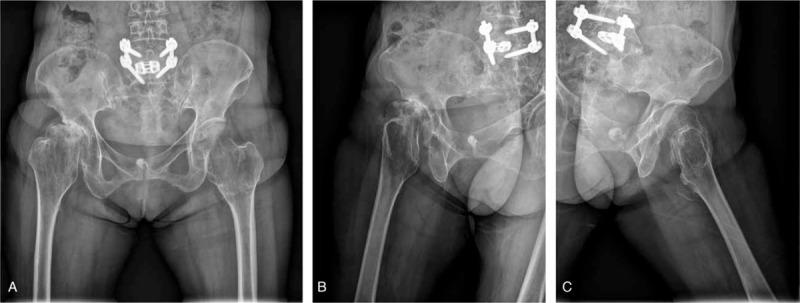
Preoperative plain anteroposterior radiograph (A), and right and left lateral (B and C) radiographs showing metaphyseal bulging with a coxa valga deformity of the bilateral proximal femur and osteoarthritic changes in the right femoro-acetabular joint.

**Figure 2:**
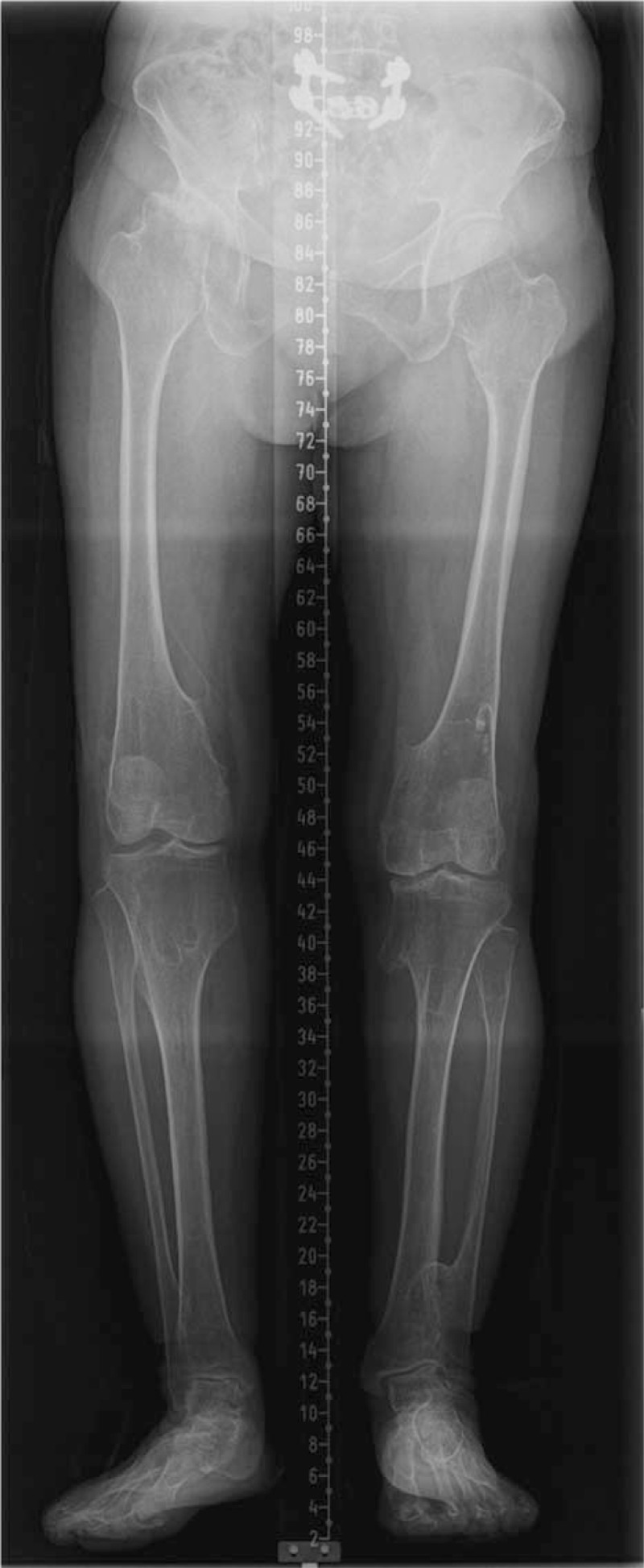
Preoperative lower extremity anteroposterior scanogram showing a limb length discrepancy with pelvic tilt.

**Figure 3:**
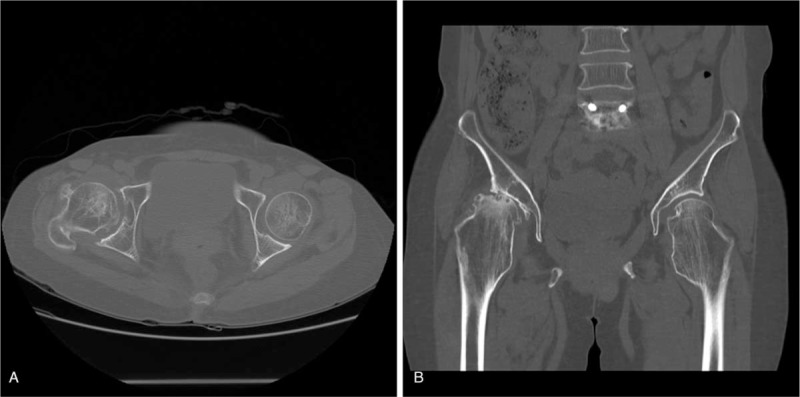
Preoperative axial (A) and coronal (B) images from a computed tomography scan confirmed multiple exostoses with a cartilaginous cap and severe osteoarthritic changes in the right hip joint.

A plain radiograph showed metaphyseal bulging with a coxa valga deformity of the bilateral proximal femur and OA changes in the right femoro-acetabular joint (Fig. 1A–C). The femoral neck angle was 159° on the right side and 155° on the left side. The femoral head on the right side was collapsed and subluxated. Multiple exostoses were detected on a lower extremity anteroposterior scanogram, and the right lower extremity was 2.4 cm longer than the left with a pelvic tilt (Fig. 2). A computed tomography scan confirmed multiple exostoses with a cartilaginous cap and severe OA changes in the right hip joint (Fig. 3A and B).

**Figure 4:**
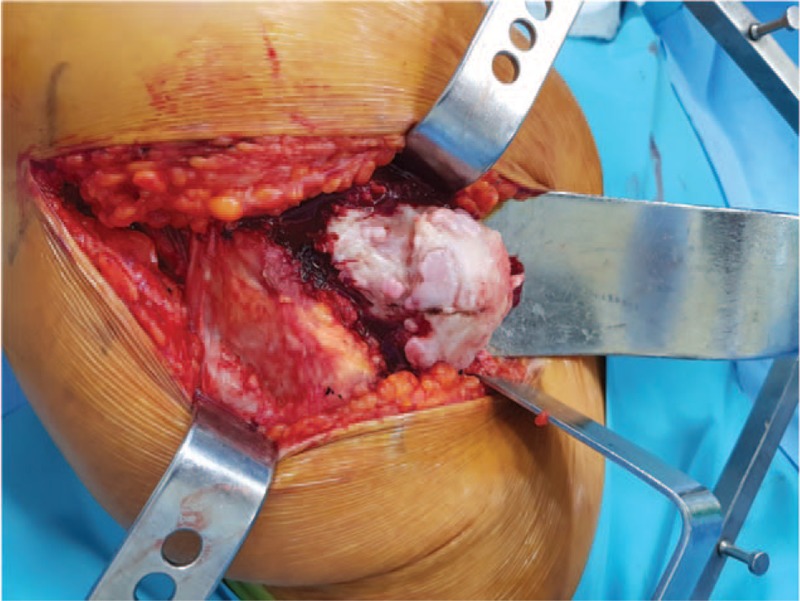
Severe articular cartilage wear and numerous osteochondromas of the femoral head and neck.

**Figure 5:**
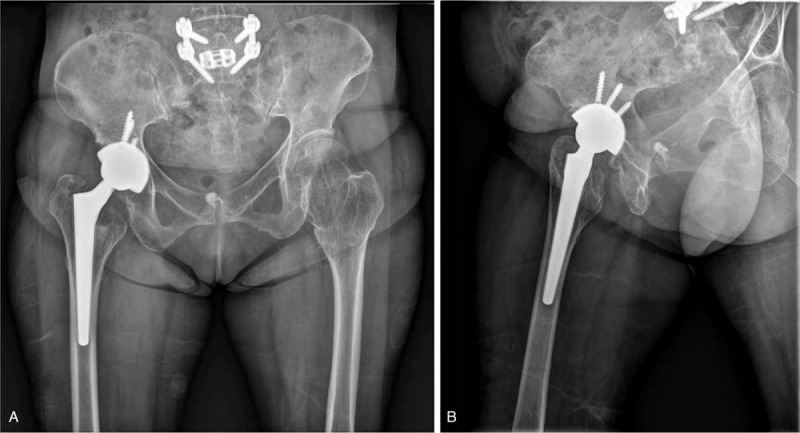
Postoperative 2-year follow-up plain anteroposterior (A) and lateral (B) radiographs showing stable conversion of the right hip joint to total hip arthroplasty.

The patient underwent total hip arthroplasty of the right hip joint. A modified posterolateral skin incision was made. The iliotibial band and gluteus maximus were split, and the short external rotator muscles were detached from the greater trochanter. A T-shaped capsulotomy was performed, and the joint was dislocated to expose the femoral head and neck. Articular cartilage wear and subchondral bone exposure of the femoral head were observed, as well as numerous osteochondromas (Fig. 4). A femoral neck osteotomy was performed at the level of the upper margin of the lesser trochanter to correct the valgus deformity and limb length discrepancy, and to graft bone around the proximal stem. The cut femoral head and neck were removed, and the metaphyseal osteochondroma was excised. All excised lesions were sent for histopathological evaluation. The hip joint was replaced with a 48 mm acetabular cup (Lima Corp., Villanova di San Daniele del Friuli, Italy), 32 mm internal diameter ceramic liner (Biolox delta; CeramTec), 32 mm ceramic head (Biolox delta; CeramTec), and a cementless Wagner cone prosthesis (Zimmer, Winterthur, Switzerland) using a 135° stem (Fig. 5A, B). The allobone graft was completed after the stem was engaged for the empty spaces in the proximal femoral metaphysis. The biopsy of the excised bony mass revealed a bony stalk with a hyaline cartilaginous cap.

Postoperative rehabilitation was carried out in the same manner as for uncomplicated total hip arthroplasty. Pain control was emphasized in the early stage after surgery, and tolerable ROM exercises were permitted at 1 week postoperatively, except internal rotation. The patient was allowed to engage in partial weight bearing ambulation as tolerated, beginning 3 days postoperatively. The patient had no limitations of daily activities and the Harris Hip Score improved from 35 preoperatively to 82 postoperatively at the 2-year follow-up.

## 3 Discussion

HME is an autosomal dominant inherited disease characterized by the development of multiple exostoses, with an incidence of 1 in 50,000.^[4,10]^ More than 90% of HME cases exhibit heterozygous germline mutations of the exostosin glycosyltransferase 1 (EXT1) and EXT2 genes.^[11]^

HME occurs primarily in the near metaphysis of the long bones. Osteochondromas cause various health complications due to their size, location, and considerable number. They are associated with growth plates, so they affect skeletal elongation and function, leading to growth retardation and malformation.^[2]^

In particular, lesions that develop around the hip joint cause painful limitations in motion due to deformities, as well as limb length discrepancies due to growth disturbances. About 25% of HME patients with hip involvement develop acetabular dysplasia, coxa valga, and a secondary deformity.^[12]^ A coxa valga deformity is a common deformity that increases the neck and shaft angle of the femur, resulting in subluxation.^[2,13]^ A large number of HME patients with hip lesions complain of pain due to impingement between the mass of the femoral neck and the acetabulum.^[14]^ El-Fiky et al^[15]^ reported that medial femur and peri-acetabular lesions affect development more so than do lateral femoral lesions, leading to coxa valga deformity and acetabular dysplasia. They suggested that subluxated hips with these deformities in symptomatic adults require surgery. An osteochondroma of the femoral neck may also cause ischiofemoral impingement with the ischium extra-articularlly.^[16]^ The deformation is irreversible in skeletally mature patients; therefore, surgical excision may be necessary for symptomatic lesions to restore joint function and lessen the pain.

Corrective osteotomy of a coxa valga deformity and excision of mass lesions can be used to prevent progressive subluxation of the hip joint without femoral head involvement, but joint replacement arthroplasty should be considered in cases of combined secondary OA. However, expansion of the proximal femoral metaphysis, particularly exostoses of the medial aspect and distortion of the normal anatomical structures, makes total hip arthroplasty more complicated and technically difficult. Care should be taken when accessing the medial part of the proximal femur, as an enlarged proximal portion is closer to the neurovascular structures than the normal anatomy. Therefore, surgeons require sufficient preoperative planning and technical acumen. In addition, the choice of implant should be carefully considered. A proximal fitted femoral stem cannot be used because there is an empty space in the proximal femur after removing expanded metaphyseal lesions. A conically designed femoral stem for press-fit fixation is more suitable in these cases. Selecting an appropriately sized femoral stem to correct a limb length discrepancy and pelvic girdle asymmetry is also very important.^[17]^ In some cases, bone grafting should be considered if there is a wide metaphyseal space.

Total hip arthroplasty could be a good clinical treatment option for patients suffering from secondary OA due to HME. Few reports have assessed surgical treatment for HME patients with symptomatic hip lesions, so in our opinion it would be important to acquire as much information as possible through review of the relevant published literature.

## 4 Conclusion

Total hip arthroplasty could be a good clinical treatment option for patients suffering from secondary OA due to HME. Surgeons must consider preoperative planning for deformities that may occur in HME patients, proceed carefully and with sufficient understanding of anatomy, and select the appropriate implant to correct deformities and prevent complications.

## Author contributions

**Conceptualization:** Woo Jong Kim, Hong Seop Lee, Byung-Woong Jang.

**Data curation:** Chang Hyun Kim, Sung Hun Won, Hong Seop Lee, Dhong Won Lee, Jae Jun Lee, Jin Ku Kang.

**Resources:** Dhong Won Lee.

**Supervision:** Jong Seok Park, Jin Ku Kang.

**Writing – original draft:** Chang Hyun Kim, Byung-Woong Jang, Yong Cheol Hong, Ricky wibowo.

**Writing – review & editing:** Woo Jong Kim, Jong Seok Park, Sung Hun Won.

Chang Hyun Kim orcid: 0000-0002-2539-1058.
